# Correction of skeletal class II severe open bite with temporomandibular joint disorder treated by miniscrew anchorage and molar extraction: a case report

**DOI:** 10.1186/s13256-019-2132-6

**Published:** 2019-07-07

**Authors:** Masato Kaku, Taeko Yamamoto, Yuka Yashima, Jin Izumino, Haruka Kagawa, Kazutaka Ikeda, Kotaro Tanimoto

**Affiliations:** Department of Orthodontics and Craniofacial Developmental Biology, Hiroshima University Graduate School of Biomedical and Health Sciences, 1-2-3 Kasumi, Minami-ku, Hiroshima, 734-8553 Japan

**Keywords:** Open bite, Skeletal class II, Steep mandible, TMD, DDwoR, Stabilization splint, Orthodontic treatment, Miniscrew

## Abstract

**Background:**

Little information is available on the treatment of open bite with temporomandibular joint disorder by intrusion of molars using miniscrews.

**Case presentation:**

This case report describes a 42-year-old Japanese woman with a skeletal class II severe anterior open bite and temporomandibular joint disorder. The pretreatment magnetic resonance imaging of both temporomandibular joints revealed osteoarthritis and anterior disc displacement without reduction in both temporomandibular joints. A stabilization splint was used before orthodontic treatment and bilateral upper and lower premolars were extracted. Miniscrews were inserted into the palatal region to intrude the maxillary molars and avoid loss of anchorage. The maxillary left first molar was also extracted to improve the molar relationship and the dental midline. Normal overjet and overbite with Angle class I molar relationship were achieved, and the upper and lower midlines coincided. Our patient’s teeth continued to be stable and her temporomandibular joint was asymptomatic after a retention period of 2 years.

**Conclusions:**

Intrusion of molars by miniscrews is available for skeletal class II severe open bite.

## Background

Temporomandibular joint disorder (TMD) is a comprehensive term and is characterized by the clinical presentation of: pain in the masticatory musculature and in the temporomandibular joint (TMJ), limited range of mandibular movement, and clicking or crepitus during jaw movement [1]. The etiology of TMD is suggested to be multifactorial, with malocclusion being a potential risk factor [2]. Numerous treatment methods have been described for anterior disc displacement without reduction (DDwoR) of TMJ. Among them, orthodontic treatment along with an occlusal splint is considered quite effective for managing TMD with anterior disc displacement [3].

In open bite cases, overgrowth of the maxillary and mandibular posterior dentoalveolar heights is often observed [4, 5], and cases of skeletal class II open bite with a steep mandible are more difficult to treat because of the increased vertical facial height [6, 7]. Therefore, high-pull headgear with a transpalatal arch [8] is traditionally used to correct the over-erupted posterior molar regions. However, this approach of reducing the posterior dentoalveolar height using headgear is not always effective as the treatment outcome is greatly influenced by the patient’s cooperation. Therefore, nowadays, miniplate [9–12] and miniscrews [13–18] are used currently for absolute anchorage. Cases of anterior open bite are often associated with TMD, and only a few reports describe the management of open bite and TMD by molar intrusion using miniscrew anchorage [19–22].

In this case report, we describe the outcome of severe skeletal class II open bite treated using miniscrews along with extraction of the four premolars and the left maxillary first molar.

## Case presentation

### Pretreatment evaluation

Our patient, a Japanese woman aged 42 years and 6 months, visited our dental hospital with a chief complaint of impaired masticatory function due to anterior open bite. She also experienced pain in the TMJ while chewing and mouth opening. Her open bite had worsened gradually and she also had tongue thrust. She was previously recommended orthodontic treatment with orthognathic surgery by an orthodontist, but she did not want to undergo the surgery.

Her pretreatment facial appearance revealed a convex profile, and suggested hypermentalis activity associated with lip closure. The initial intraoral photographs revealed a − 6.0-mm anterior open bite and occlusal contact between only the second molars. The right molar occlusal relationship was class II, while that of the left was a more severe type of class II. There was crowding in either arch and the upper dental midline had shifted to the right by 4 mm. The lower midline coincided with the facial midline (Figs. 1 and 2). A panoramic radiograph revealed the existence of three third molars except the upper right third molar (Fig. 3). A severe skeletal class II relationship of angle of point A-nasion-point B (ANB), 11.5° and a steep Frankfort-mandibular plane angle (FMA, 47.5°) with lingual inclination of upper incisors of upper incisor-Frankfort plane angle (U1-FH), 104.6° was noted in the cephalometric measurements (Table 1). The DDwoR of both TMJs was evident in the magnetic resonance imaging (MRI) images (Fig. 4a, b). Schüller’s view showed flattening of both condyles, but there was no restriction of jaw movement (Fig. 5a, b). Based on this information, our patient was diagnosed to have a skeletal class II open bite with TMD. Informed consent was obtained from all individual participants included in the study.

**Fig. 1:**
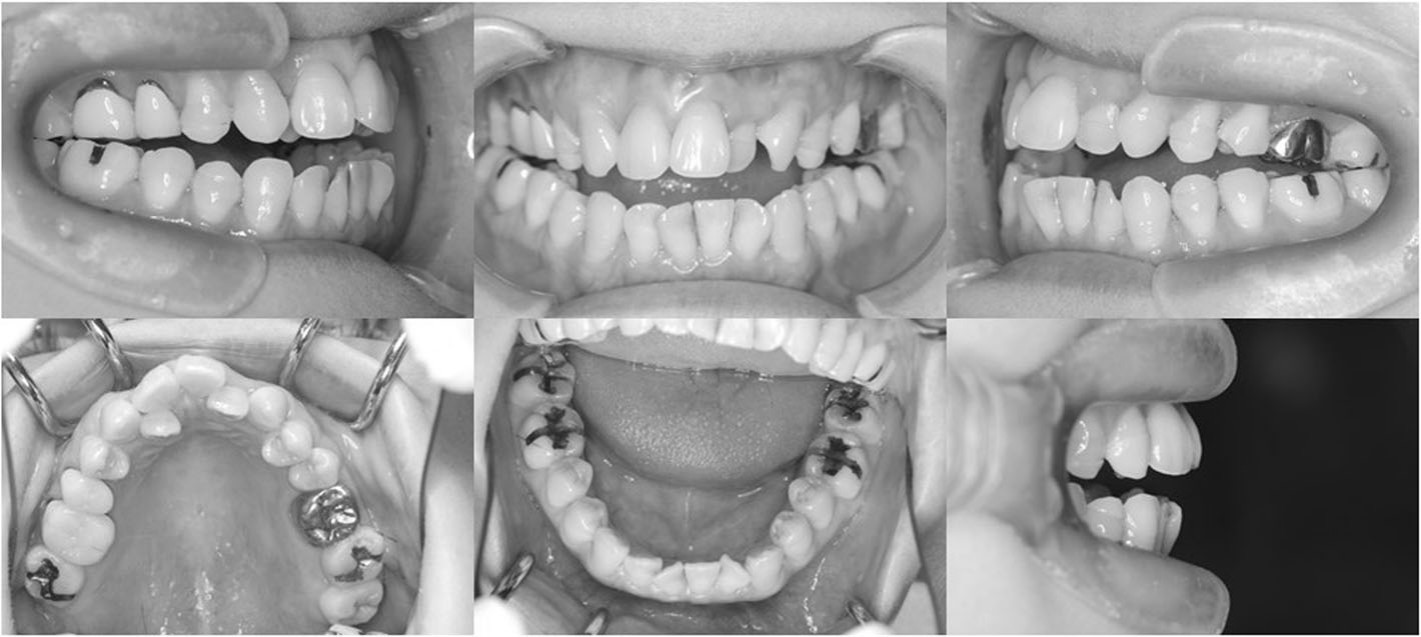
Pretreatment intraoral photographs

**Fig. 2:**
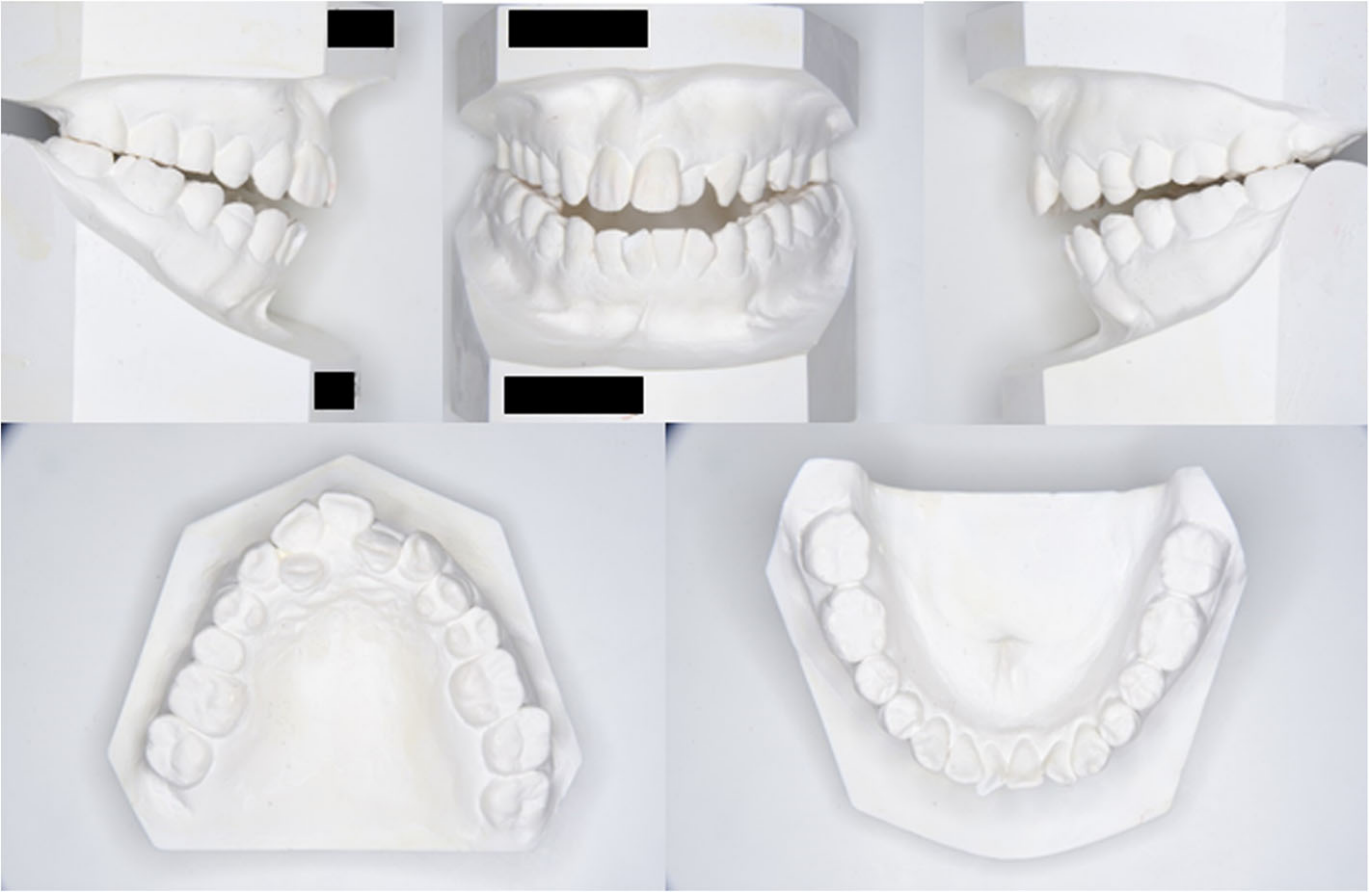
Pretreatment dental casts

**Fig. 3:**
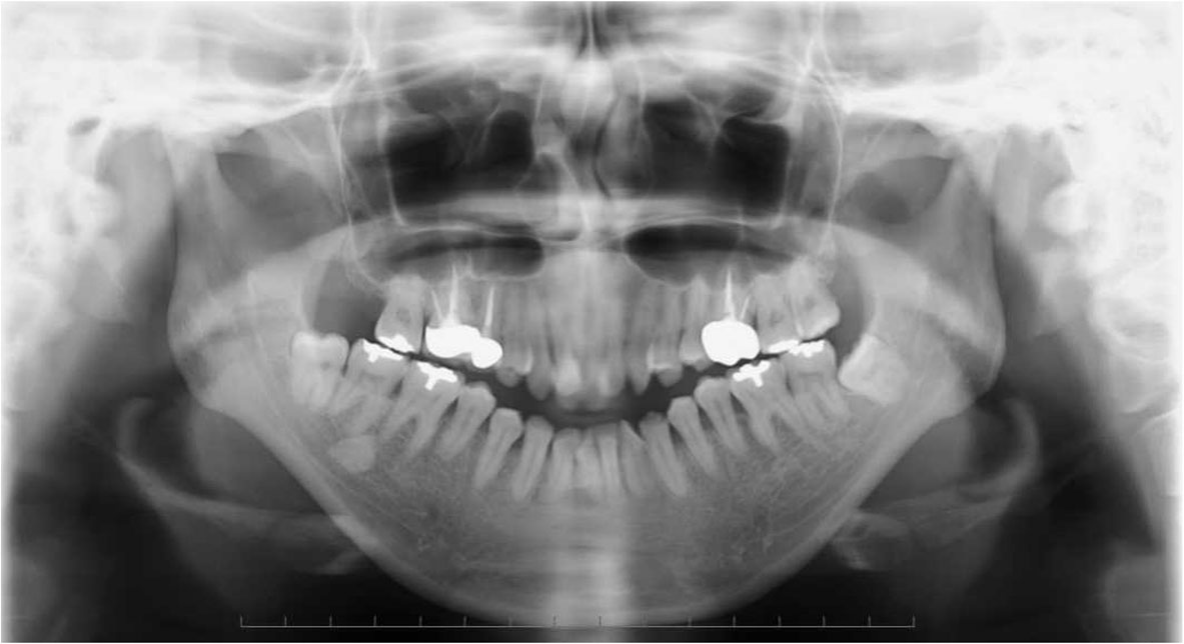
Pretreatment panoramic radiograph

**Table 1:** Summary of cephalometric measurements

Measurements	Pre-treatment	Post-treatment
SNA (°)	80.0	79.3
SNB (°)	68.4	68.8
ANB (°)	11.5	10.1
FMA (°)	47.5	46.5
FMIA (°)	40.5	53.2
IMPA (°)	92.0	80.3
U1-FH (°)	104.6	96.2
Over jet (mm)	3.5	1.5
Over bite (mm)	-6.0	1.5

*SNA* angle of sella-nasion-point A, *SNB* angle of sella-nasion-point B, *ANB* angle of point A-nasion-point B, *FMA* Frankfort-mandibular plane angle, *FMIA* Frankfort-mandibular incisor angle, *IMPA* incisor mandibular plane angle, *U1-FH* upper incisor-Frankfort plane angle, *(°)* measurement of a plane angle

**Fig. 4:**
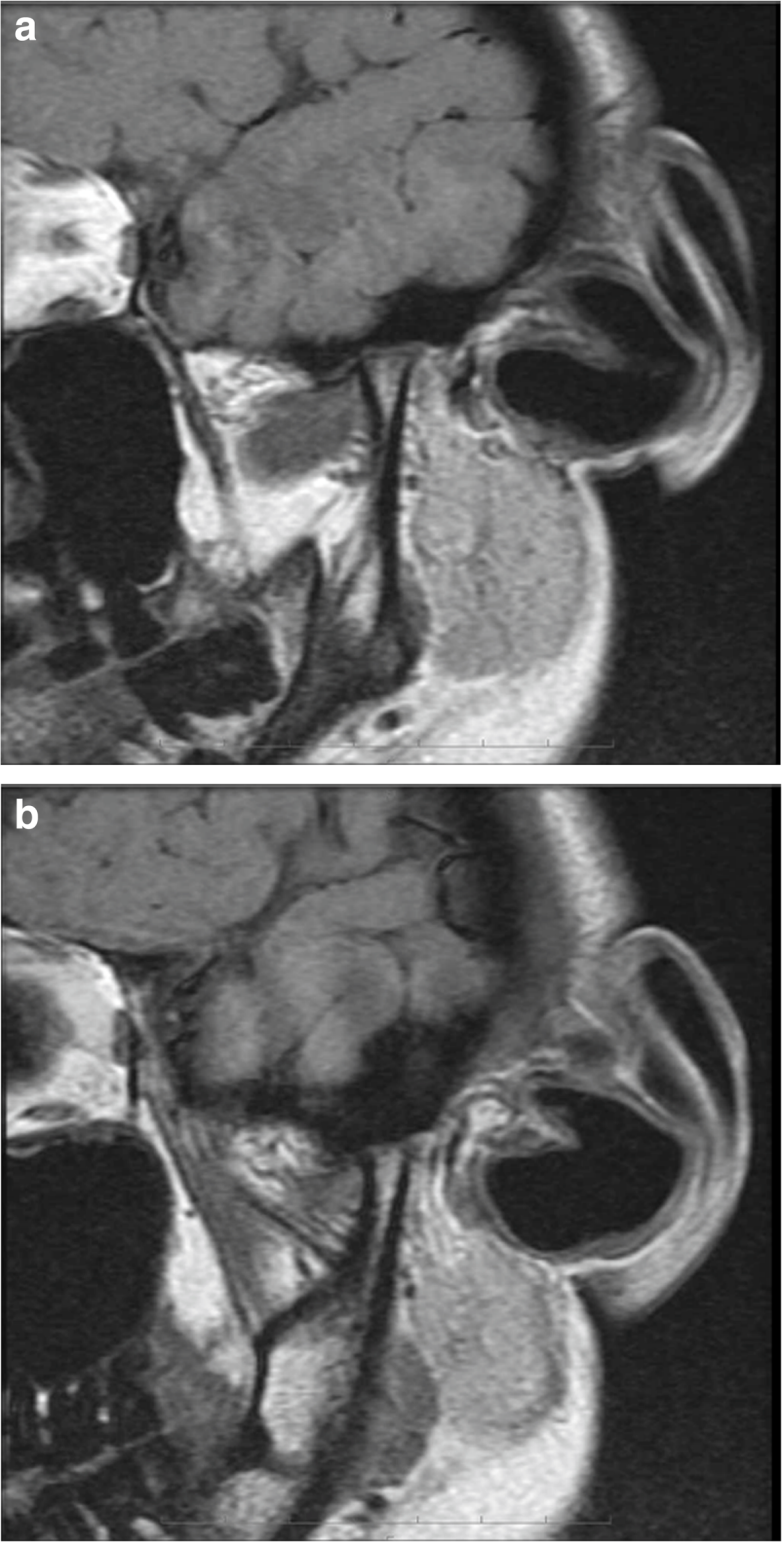
Pretreatment magnetic resonance imaging. **a** Right side; **b** left side

**Fig. 5:**
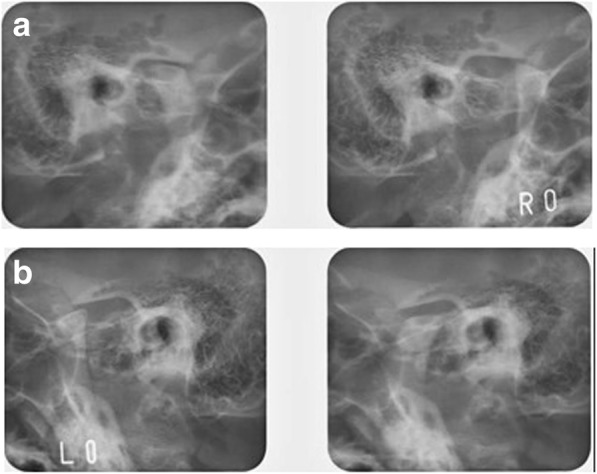
Pretreatment Schüller’s view. **a** Right side central occlusion and open; **b** left side central occlusion and open

### Treatment plan

A stabilization occlusal splint was used before the orthodontic treatment to reduce the TMJ pain on masticatory movement. Because our patient refused surgical treatment, it was decided to correct the anterior open bite and achieve an ideal occlusion with class I molar relationship via orthodontic treatment alone (without orthognathic surgery). The treatment plan was as follows.

1. Extraction of the maxillary right and left first premolars
2. Extraction of the mandibular right and left second premolars.
3. Insertion of miniscrews into the palatal region and left alveolar bone of mesial part of first molars to intrude the maxillary molars and to avoid anchorage loss.
4. Correction of crowding and distal movement of anterior teeth.
5. Extraction of the maxillary left first molar to correct the midline and left molar relationship.
6. Preparation and insertion of a retainer with tongue crib to avoid tongue thrust.

### Treatment progress

Before the orthodontic treatment, a stabilization occlusal splint was placed for 3 months to reduce the TMJ pain. After our patient confirmed alleviation of TMD symptoms, the four premolars were extracted and 0.018-inch (0.457-mm) standard edgewise brackets were bonded on the maxillary and mandibular teeth. This was followed by placement of two self-drilling titanium alloy miniscrews (2.0 mm in diameter and 6 mm in length, Dual Top Auto Screw; Jeil Medical Corp., South Korea) into the palatal region of the maxillary first molar, under local anesthesia, to intrude the maxillary posterior teeth. A transpalatal arch was also placed in order to maintain the maxillary molar width (Fig. 6). The molars were connected by an elastic chain to intrude them and to avoid the loss of anchorage. Distal movement of the maxillary canines and mandibular first premolar was then initiated.

**Fig. 6:**
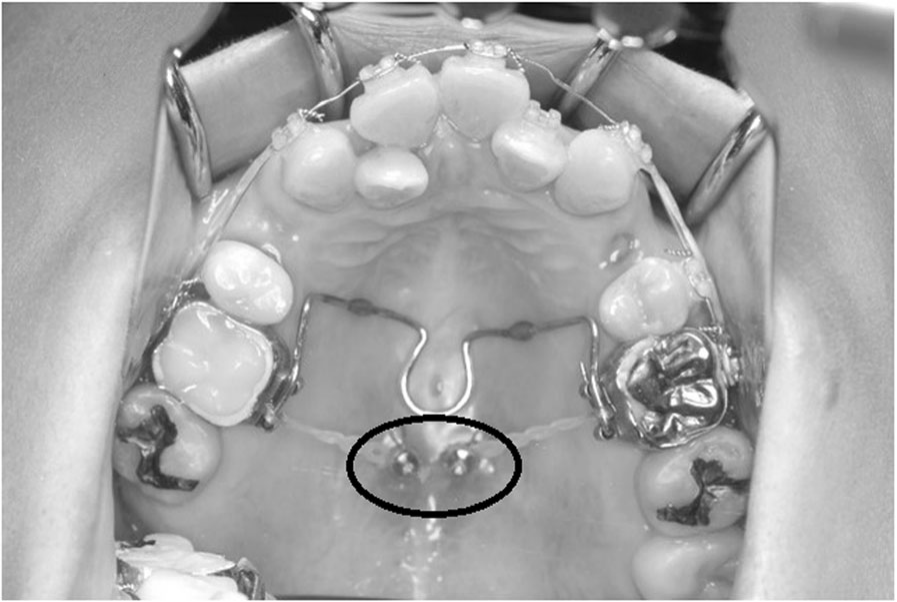
Intrusion of the molars by elastic chain. A *circle* shows miniscrews

After 24 months, the extraction spaces of premolars had closed. The maxillary dental midline had shifted to the right by 3 mm. However, the left canine and molar relationship continued to be class II, while the right side had nearly progressed to a class I relation (Fig. 7). Hence, the maxillary left first molar was extracted to correct the midline and the left molar relationship. The maxillary left premolars were moved distally using a miniscrew (1.6 mm in diameter and 8 mm in length) which was inserted into the maxillary left buccal alveolar bone.

**Fig. 7:**
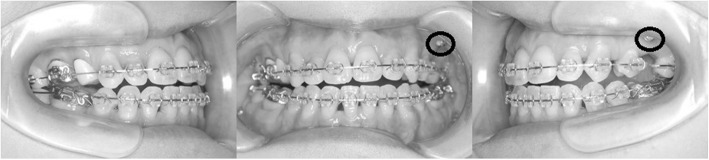
Intraoral photographs during treatment (after extraction of the upper left first molar, a *circle* shows a miniscrew)

After 36 months, the anterior open bite was corrected to 1.5 mm and the teeth had attained a class I molar relationship (Figs. 8 and 9). All the orthodontic appliances and miniscrews were then removed, and lingual bonded retainers were affixed on both arches. In addition, a Begg-type retainer with tongue crib was also affixed in the maxilla to avoid tongue thrust. Our patient did not have any TMD symptoms during the active treatment and the retention period. The occlusion continued to be stable at 24 months from the initiation of the retention.

**Fig. 8:**
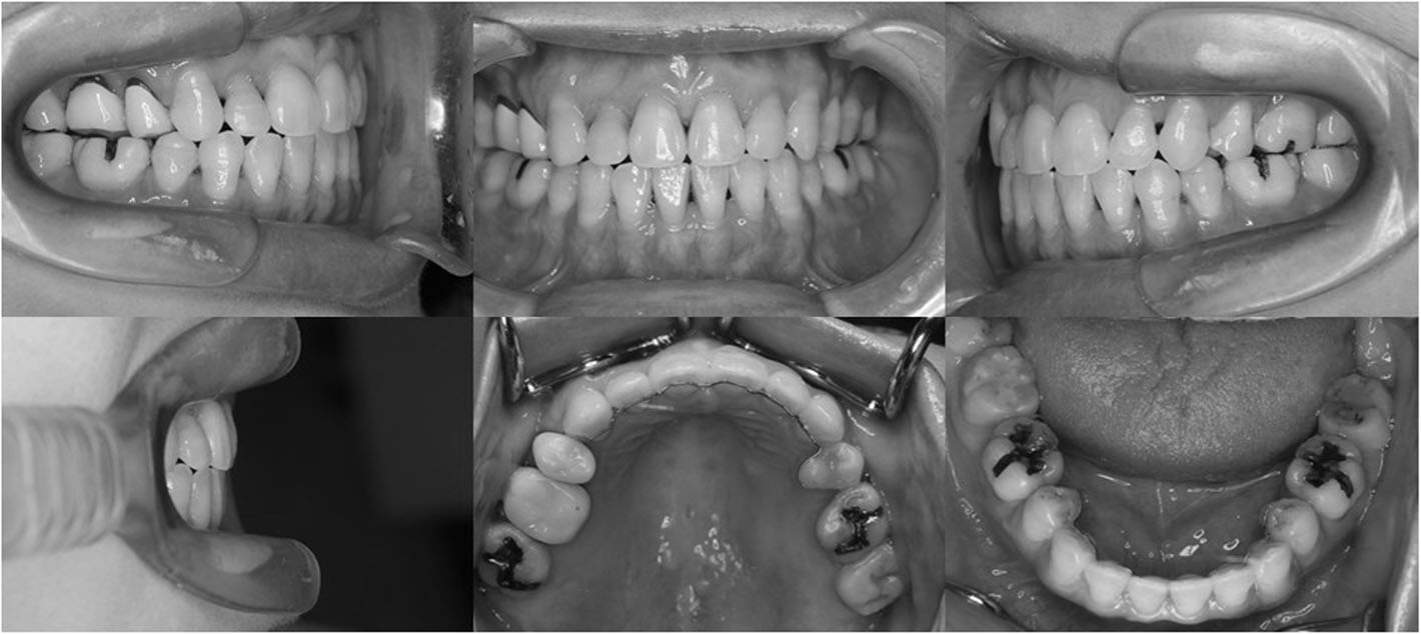
Posttreatment intraoral photographs

**Fig. 9:**
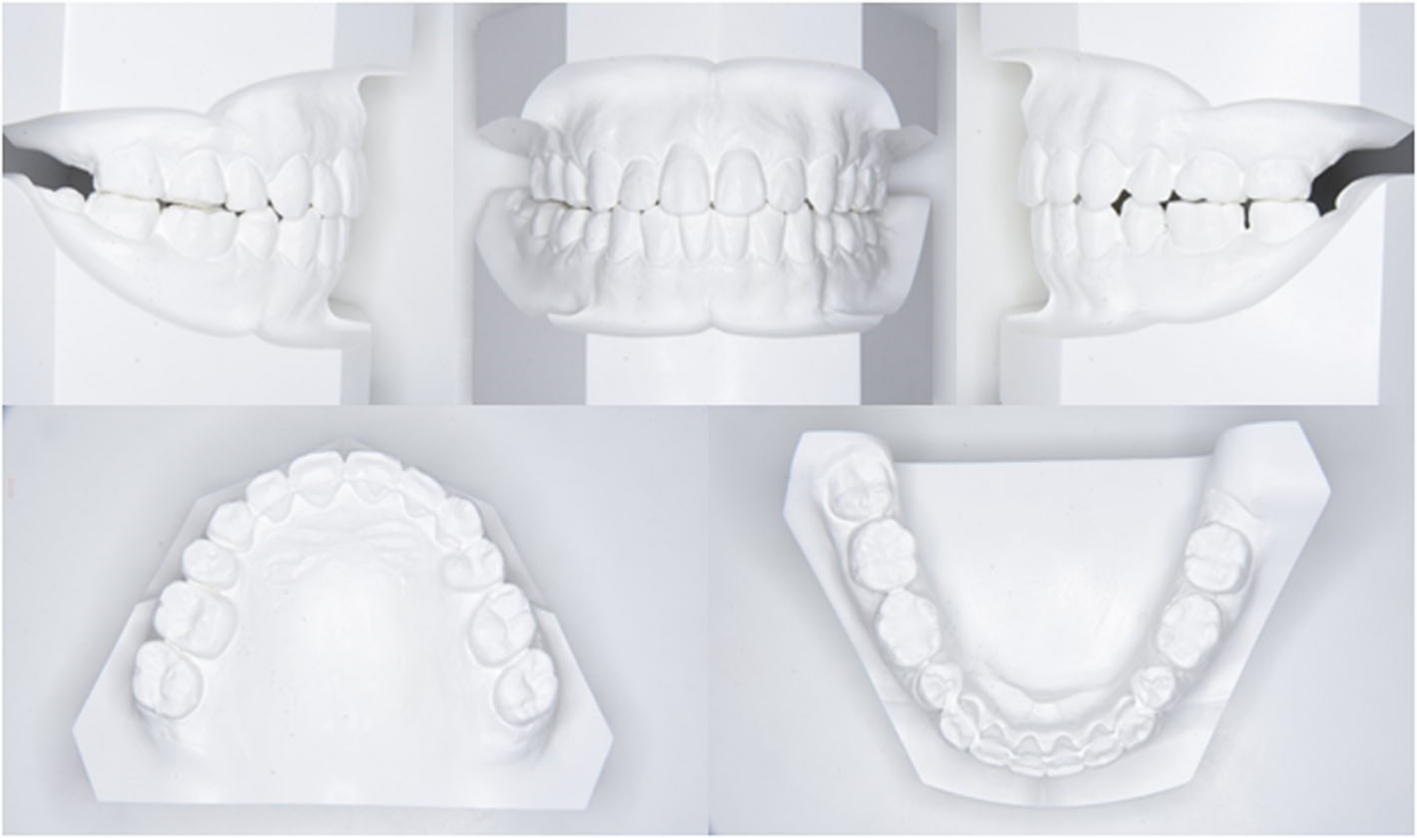
Posttreatment dental casts

### Treatment results

After orthodontic treatment, the overbite increased to 1.5 mm, while the molar and canine relationships changed to class I on both sides. The maxillary and mandibular dental midlines coincided, and the arch alignment was well corrected. There was no major variation in the posttreatment facial profile compared with the pretreatment profile. Although, the posttreatment panoramic radiograph suggested minor root resorption of the maxillary incisors, all roots were aligned in parallel (Fig. 10). On the cephalometric pretreatment and posttreatment superimposition, the upper and lower anterior teeth were distalized by 5 mm and 3 mm, respectively, and extruded by 2 mm and 3 mm, respectively. The maxillary molars were intruded by 1 mm (Fig. 11). The ANB angle had changed from 11.5° to 10.1°, and the FMA had changed from 47.5° to 46.5° (Table 1). Posttreatment Schüller’s view revealed no change in condyle shape and jaw movement (Fig. 12a, b). Our patient did not experience any symptoms of TMD, such as pain on mouth opening, during the orthodontic treatment.

**Fig. 10:**
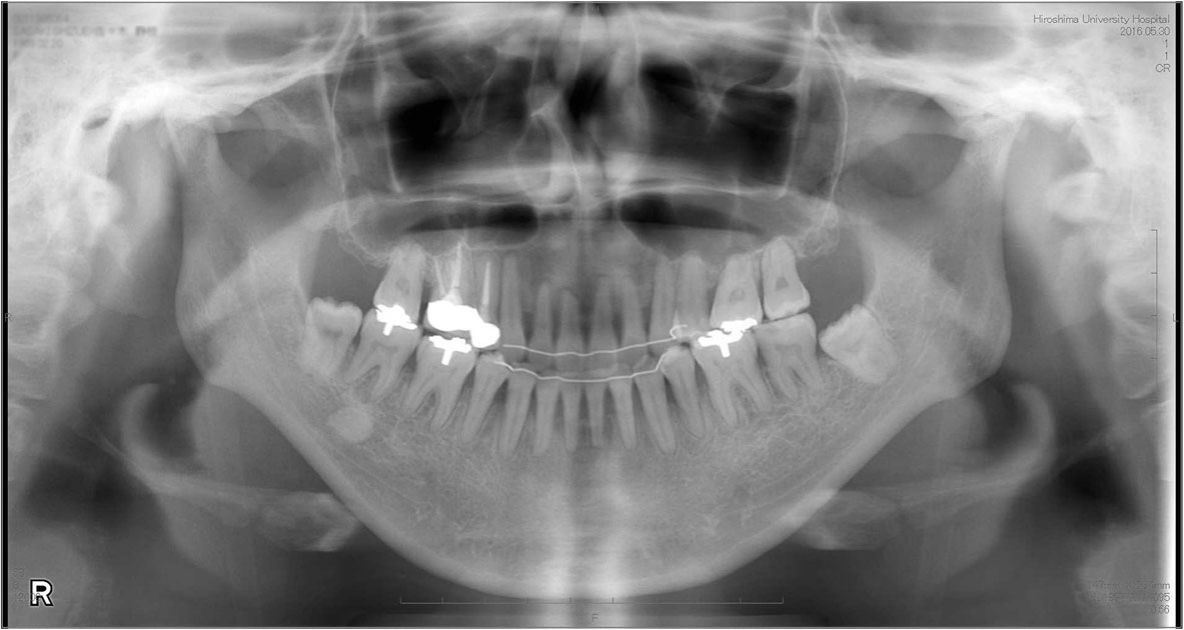
Posttreatment panoramic radiograph

**Fig. 11:**
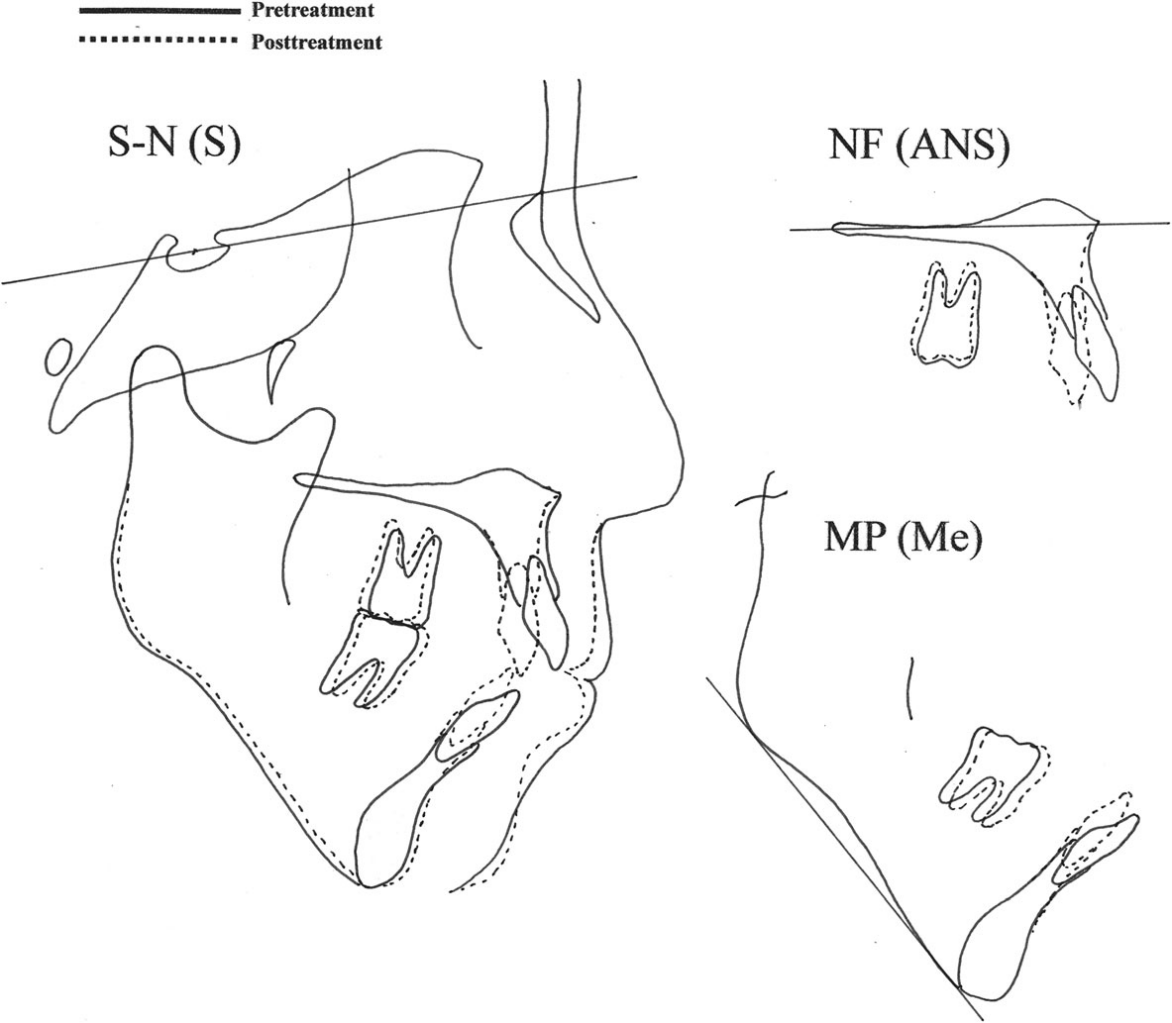
Cephalometric superimposition (pretreatment and posttreatment). *MP* (*Me*) superimposition for mandibular plane at menton, *NF* (*ANS*) superimposition for nasal floor at anterior nasal spine, *S-N* (*S*) superimposition for sella-nasion at sella

**Fig. 12:**
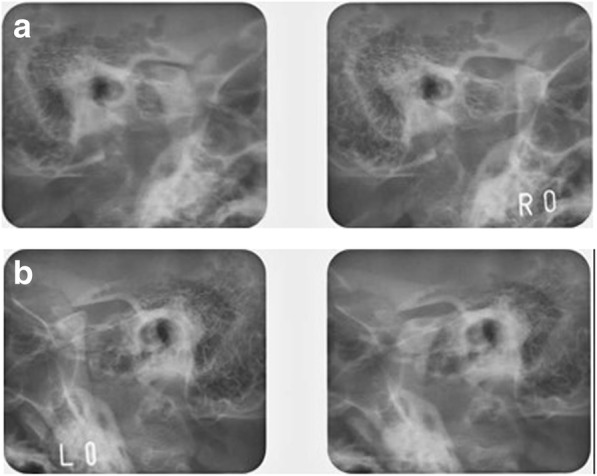
Posttreatment Schüller’s view. **a** Right side central occlusion and open; **b** left side central occlusion and open

## Discussion

The TMJ disc in patients with DDwoR is shifted anteriorly and cannot revert to the correct position during jaw movement, resulting in TMJ pain and limitation of jaw movement [23]. Numerous treatment methods have been tried for managing DDwoR including manipulation, internal medicine [24], and surgical correction [25]. Although the exact mechanism of the occlusal splint is not clear [26], it is one of the important and frequently used treatment modalities. It has been suggested that splint therapy may reduce overloading on the TMJ and relieve the masticatory muscles [24]. A 2-year follow-up study suggested that splint therapy effectively improved the maximum mouth opening and alleviated pain associated with DDwoR [27]. The study by Stiesch-Scholz *et al*. also suggested that stabilization and pivot splints improved maximum mouth opening and reduced TMJ pain related to DDwoR [28].

In this case, our patient was diagnosed as having DDwoR via MRI, and a stabilization occlusal splint was used before orthodontic treatment to reduce the TMJ pain associated with masticatory movement. As a result of splint therapy for 3 months, the TMJ pain associated with chewing and mouth opening was relieved. There were no symptoms of TMD during the active orthodontic treatment and the retention period. Schüller’s view also revealed that there was no change of condyle shape and jaw movement before and after orthodontic treatment. However, a recent study showed that splint therapy can be continued during the first several months with orthodontic treatment by adjustment of the splint according to the tooth movement [29]. So, simultaneous recovery in the TMJ with the orthodontic treatment might be achieved without delay of the treatment in this case.

Anterior open bite can occur following overgrowth of the posterior dentoalveolar heights in the maxilla and mandible. Orthognathic surgery is considered effective in improving occlusion and facial profile in patients with severe skeletal open bite along with excessive lower facial height. In such cases, maxillary surgical impaction is often applied for the mandibular counterclockwise rotation. Le Fort I and bilateral sagittal split ramus osteotomy (SSRO) reportedly offer successful and stable outcomes in patients with skeletal open bite [30]. Hoppenreijs *et al*. reported that Le Fort I osteotomy with or without bilateral SSRO exhibited good skeletal stability in patients with skeletal anterior open bites [31]. In the present case, our patient showed a severe anterior open bite with DDwoR. Because Aghabeigi *et al*. reported that orthognathic surgery did not have any effect on TMD in patients with anterior open bite [32], and we wanted to reduce the burden on TMJ induced by orthognathic surgery, an orthodontic camouflage treatment was chosen in this patient. However, Thilander *et al*. suggested that orthognathic surgery was effective in improving the symptoms of TMD [33]; study of the role of orthognathic surgery in the management of TMD should be progressed.

A previous study showed that molar intrusion by miniscrew anchorage was an effective treatment option in patients with TMD who have horizontal open bite with a steep mandible [20]; hence, this option was chosen in the current study to correct the anterior open bite via orthodontic treatment using miniscrew anchorage. Xun *et al.* showed that miniscrews can intrude both upper and lower molars by an average of 1.8 mm and 1.2 mm respectively which leads to a counterclockwise rotation of the mandible [34]. In the present case, the maxillary molars were intruded by 1 mm and FMA decreased by 0.8°. However, since the change was negligible, improvement of the overbite was brought about by extrusion of both maxillary and mandibular incisors.

A right shift in the maxillary dental midline was noted after the premolar extraction space was closed, while the left canine and molar relationship remained class II. Hence, the maxillary left first molar was extracted. In such cases, the third molar is removed and the second and first molars are moved distally to achieve a class I molar relationship. However, molar distalization requires more time and out patient’s left first molar was pulpless and was restored by a full-cast crown. Moreover, the left third molar was intact and the size was appropriate. Hence, it was decided to extract the first molar to correct the midline and left molar relationship. As a result, a good intercuspal relationship with a normal overjet and overbite were achieved and the maxillary and mandibular midlines coincided. However, since long-term stability of open bite correction depends on many factors, such as tongue thrust, periodic checkups and examinations of the TMJ are necessary for this patient.

## Conclusions

1. Severe open bite with a class II molar relationship can be treated with miniscrews and molar extraction.
2. Occlusal splint therapy and orthodontic treatment are useful for managing patients with DDwoR.
3. The authors have no conflicts of interest directly relevant to the content of this article.

## Data Availability

Not applicable.

## Abbreviations

ANB: Angle of point A-nasion-point B
DDwoR: Anterior disc displacement without reduction
FMA: Frankfort-mandibular plane angle
MRI: Magnetic resonance imaging
SSRO: Sagittal split ramus osteotomy
TMD: Temporomandibular joint disorder
TMJ: Temporomandibular joint
